# GHOSTX: An Improved Sequence Homology Search Algorithm Using a Query Suffix Array and a Database Suffix Array

**DOI:** 10.1371/journal.pone.0103833

**Published:** 2014-08-06

**Authors:** Shuji Suzuki, Masanori Kakuta, Takashi Ishida, Yutaka Akiyama

**Affiliations:** Graduate School of Information Science and Engineering, Tokyo Institute of Technology, Meguro-ku, Tokyo, Japan; American University in Cairo, Egypt

## Abstract

DNA sequences are translated into protein coding sequences and then further assigned to protein families in metagenomic analyses, because of the need for sensitivity. However, huge amounts of sequence data create the problem that even general homology search analyses using BLASTX become difficult in terms of computational cost. We designed a new homology search algorithm that finds seed sequences based on the suffix arrays of a query and a database, and have implemented it as GHOSTX. GHOSTX achieved approximately 131–165 times acceleration over a BLASTX search at similar levels of sensitivity. GHOSTX is distributed under the BSD 2-clause license and is available for download at http://www.bi.cs.titech.ac.jp/ghostx/. Currently, sequencing technology continues to improve, and sequencers are increasingly producing larger and larger quantities of data. This explosion of sequence data makes computational analysis with contemporary tools more difficult. We offer this tool as a potential solution to this problem.

## Introduction

Protein sequence homology searches are essential for identifying potential functions, structures and evolutionary relationships. Both database sizes and the number of queries have increased rapidly in recent years, because of improvements in sequencing technology, and with so much more data, searching takes even longer. DNA sequences are usually translated into protein coding sequences and then further assigned to protein families using a homology search in metagenomic analyses, because of the need for sensitivity [1], [2]. The homology search step has become one of the major bottlenecks of the analysis. BLAST [3], [4] is a widely used homology search tool that uses a heuristic algorithm. However, the search speed of BLAST is becoming insufficient for current demands of sequence homology searches. To solve this problem, a number of tools have been developed. BLAT [5] is one of the most famous tools, and is approximately 50 times faster than BLAST. However, its search sensitivity is much lower than BLAST.

Recently, Ye et al. developed a faster and more sensitive homology search tool, RAPSearch [6], [7]. RAPSearch is approximately 20–90 times faster than BLAST, and has higher search sensitivity than BLAT. However, RAPSearch uses a reduced amino acid alphabet of ten symbols to restrict the seed sequence search space. Therefore, RAPSearch cannot use any score matrices except BLOSUM62, because the reduced amino acid alphabet is only optimized for homology searches with BLOSUM62. Thus, changing the score matrix is difficult with RAPSearch.

Here, we have developed a new, fast algorithm using suffix arrays [8] of both queries and database sequences for its seed search process. We used a seed search method relying on a score-based optimal length. In the algorithm, only seeds with a sufficient match score are searched, based on a given score matrix. Thus, the algorithm can effectively exclude seeds with sufficient length but insufficient match scores. We implemented this algorithm as GHOSTX. GHOSTX was implemented in C++ and supported on Intel CPUs with GCC (version 4 or later) and SPARC64 (VIIIfx or later) with the Fujitsu C++ compiler. It is distributed under the BSD 2-clause license and is available for download at http://www.bi.cs.titech.ac.jp/ghostx/.

## Materials and Methods

### Overview of the GHOSTX algorithm

**Figure 1:**
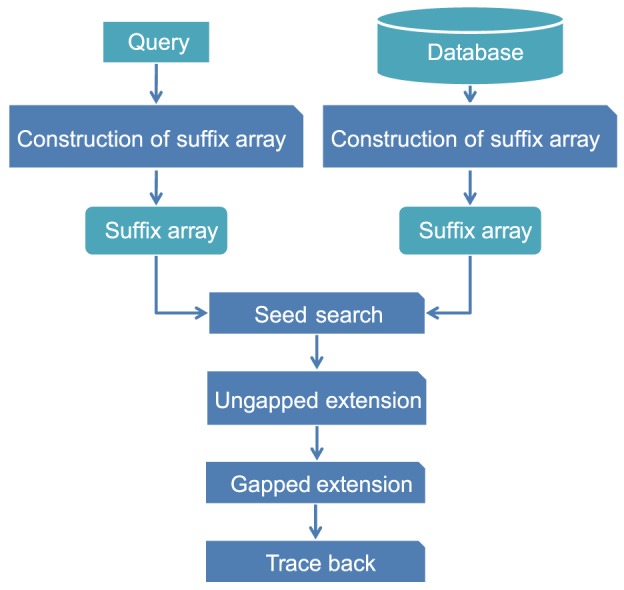
The flow of GHOSTX.

GHOSTX adopts the seed-extension approach used in BLAST. GHOSTX consists of three main steps: a seed search, an ungapped extension, and a gapped extension. The flow of GHOSTX is shown in Figure 1. Initially, GHOSTX finds seeds that are substrings of database sequences similar to the substrings of a query sequence. Next, GHOSTX makes alignments by extending those seeds without gaps, and then similar, nearby seeds are brought together by a chain filter. Finally, GHOSTX makes alignments from seeds with gaps. The gapped extension step requires heavy calculation, but the BLAST algorithm efficiently decreases the number of gapped extension candidates through its seed search and ungapped extension steps. As a result, the seed search and the ungapped extension steps are the most computationally intensive parts of BLAST. The seed search and the ungapped extension steps consume approximately 75% of the computation time of BLAST, while approximately 20% of the time is spent on the gapped extension [9]. Thus, reducing the computation time for the seed search and ungapped extension steps is effective for achieving acceleration. To accelerate the search seed step, GHOSTX uses suffix arrays for both the query sequences and the database sequences.

Our seed search method using a suffix array effectively reduces the computation time of the seed search step. As a result, the ungapped extension step then becomes the bottleneck. Thus, for further acceleration, we have to decrease the number of ungapped extensions. It would be easy to decrease the number of ungapped extension candidates by using longer seeds. However, if this is done, significant matches can be missed, and search sensitivity becomes lower. Consequentially, a sophisticated method is required for accelerating search speed, while still maintaining search sensitivity. Therefore, GHOSTX does not fix the length of a seed in the seed search step, but rather it extends the length until the matching score exceeds a given threshold. In comparison, BLAST searches with seeds of fixed lengths, and if one seed is discovered near another, BLAST performs ungapped extensions around it. BLAST seed hits with low matching scores using fixed length seeds, such as an exact match of “AAA,” whose score is only 12 based on the BLOSUM62 score matrix, are treated equally with seed hits with high matching scores, such as an exact match of “WWW,” whose score is 33. However, hits with lower scores tend to be false. Consequently, GHOSTX extends such seeds to check whether they are reliable, thus GHOSTX can use a higher score threshold than BLAST, without losing its search sensitivity. As a result, GHOSTX can reduce the number of ungapped extensions and gapped extensions needed, thereby reducing computation time after the initial seed search step.

### Suffix Array

A suffix array is the list of indexes of all suffixes of a string in a lexicographically sorted order. A suffix array can be constructed in linear time. A text *T*[0,*n*] = *t*_0_…*t_n_*_-1_ is a sequence of symbols and the length of *T* is |*T*| = *n*. Each symbol is an element of an alphabet Σ (|Σ| of protein is 20). *T*[*i*] = *t_i_* and *T*[*i*, *i*+*j*] = *t_i_*…*t_i_*_+*j*-1_ are substrings. The suffix array of *T* is *SA*, that is, an array of pointers to all the suffixes of *T* in lexicographical order. Therefore, if *i*<*j*, then *T*[*SA*[*i*]]<*T*[*SA*[*j*]]. An exact search based on a binary search for pattern, whose length is *m*, can be performed as *O*(*m*log(*n*)) with the suffix array of *T*.

### Seed Search

For two suffix arrays, we can find all the local matches using dynamic programming [10]. However, calculating all alignments using dynamic programming requires a huge amount of computation time. In GHOSTX, therefore, we introduce two methods to prune the search space.

**Figure 2:**
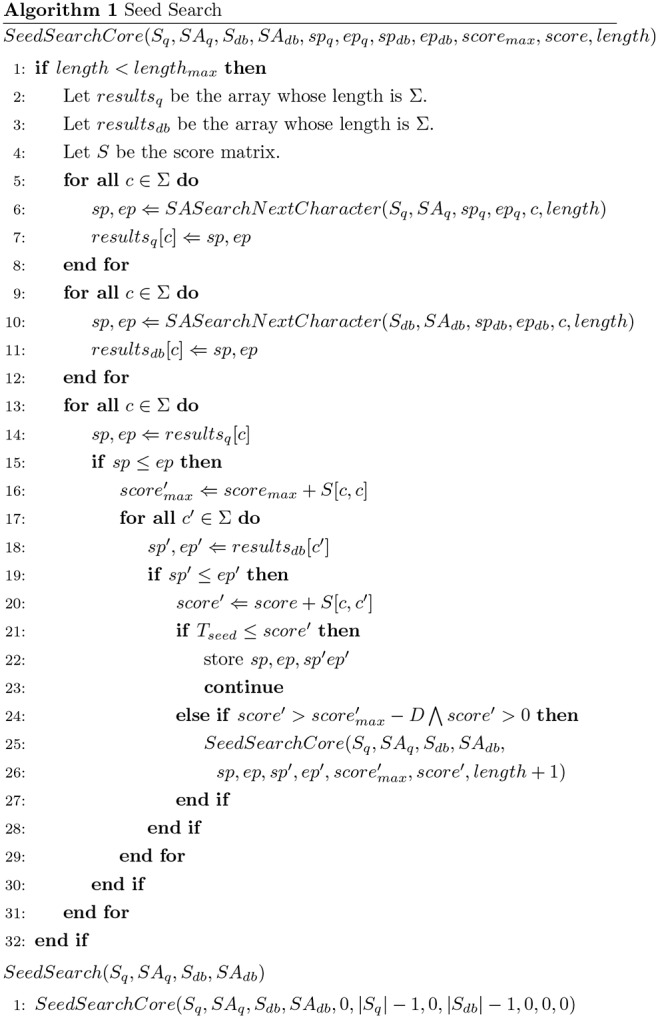
Seed search algorithm using suffix arrays.

**Figure 3:**
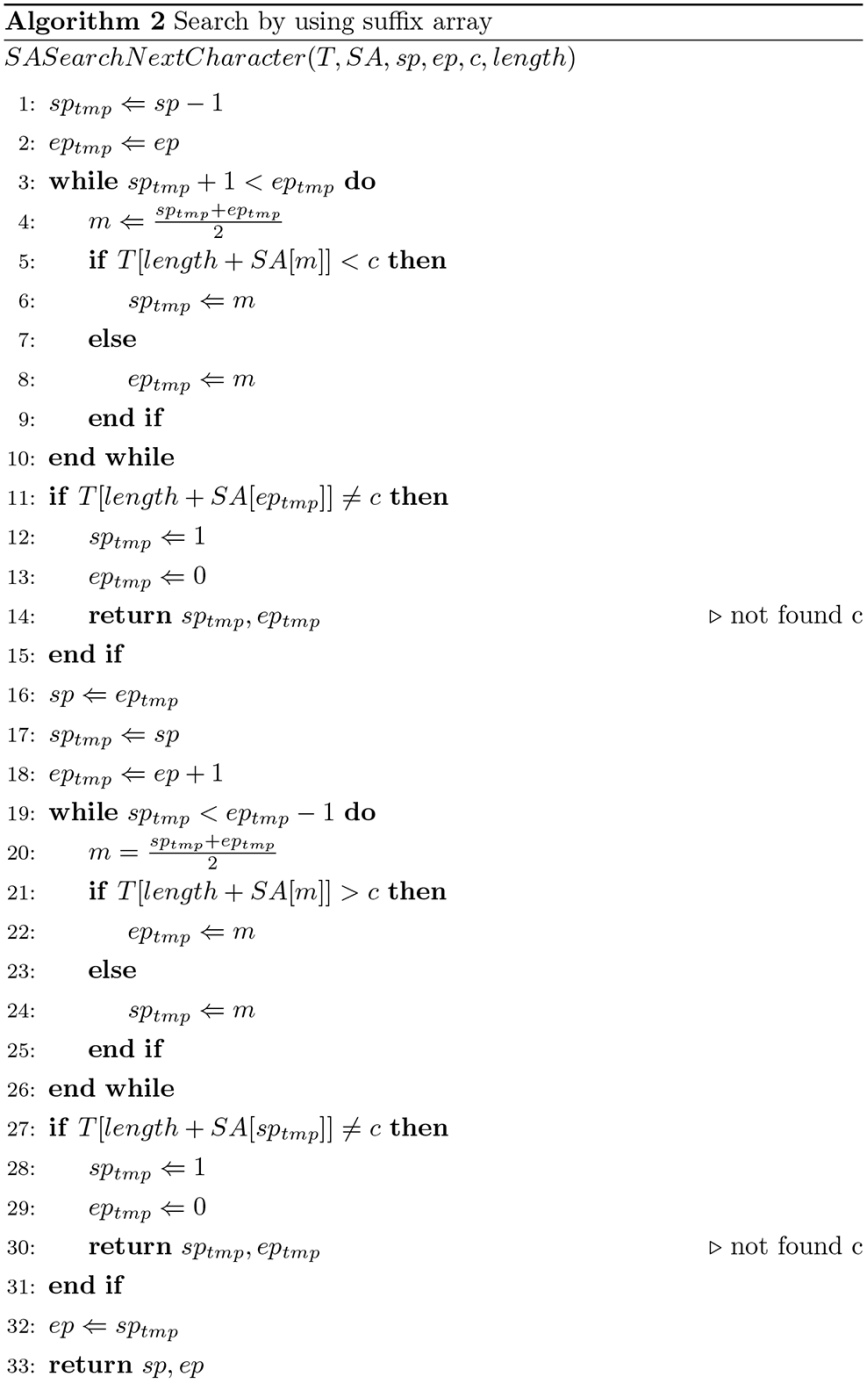
Search algorithm using a suffix array.

**Figure 4:**
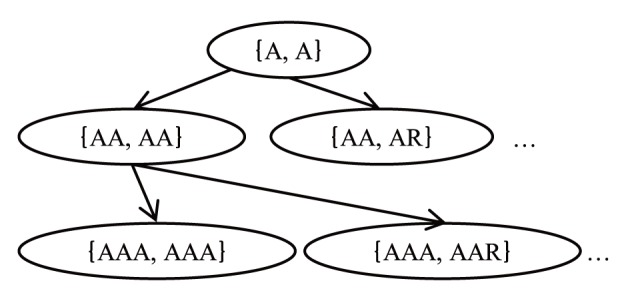
An example seed search.

Here, the sequences *S*_0_, *S*_1_,…, *S_N_*_-1_ in a database are connected with inserting delimiters to transform them into a long single sequence *S_db_* = *S*_0_#*S*_1_#…*S_N_*_-1_ (marked by the special symbol #). *SA_db_* is the suffix array of *S_db_*, and *SA_q_* is the sequence of query *S_q_*. The pair of substrings *S_db_* and *S_q_*, {*S_db_*[*i*, *i*+*l*], *S_q_*[*j*, *j*+*l*]} is the seed. Here, we want to find a seed whose score is more than the threshold *T_seed_* based on these two suffix arrays. Figure 2 shows the pseudo-code of the seed search method, and Figure 3 shows a pseudo-code for the search method of one character using a suffix array. In Figure 2, *sp_q_*, *ep_q_*, *sp_db_* and *ep_db_* are positions on *SA_q_* and *SA_db_*, and GHOSTX gets the positions of substrings from suffix arrays by using these positions. If the score of a pair of substrings {*X_db_*, *X_q_*} exceeds threshold *T_seed_*, GHOSTX keeps the pair as a seed (line 22 in Figure 2); otherwise, GHOSTX checks all pairs of extended substrings {*X_db_c’*, *X_q_c*} (*c* and *c’* are members of Σ) (line 25 in Figure 2). Thus, the maximum number of new pairs of substrings is |Σ|^2^. Using the suffix arrays of a query and a database, GHOSTX can find a substring efficiently. Figure 4 shows the example for the seed search. If {A, A} is found, GHOSTX searches the query sequence and the sequences in a database for extended substrings AA, AR, …, AV. And then, GHOSTX checks all pairs of extended substrings that are found {AA, AA}, {AA, AR}, …, {AV, AV}. GHOSTX repeats this step. However, the search takes a long time if the max seed length *length_max_* is large, because the size of the seed search space is *O*(Σ^2*length*^ *_max_*). Thus, the search space must be pruned.

GHOSTX uses two methods to prune the search space (line 24 in Figure 2). First, let *score_max_* be the sum of the exact match score of all query substring characters (line 16 in Figure 2), *score* be the score of the pair of the query and database substring (line 20 in Figure 2), and *D* be the upper limit of *score_max_* - *score*. If *score* ≤ *score_max_* - *D*, GHOSTX does not extend the substring in the pair. For example, if GHOSTX checks {AA, AR} and uses BLOSUM62 score matrix, *score_max_* of this pair is 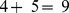 and *score* of this pair is 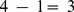. If *D* = 4. In this case, GHOSTX does not extend the substrings in this pair. Second, if the score of a substring pair is not more than 0, GHOSTX does not extend it. If *x*<*y*<*z*, the score of the substring pair {*S_db_*[*i*, *i*+*y*], *S_q_*[*j*, *j*+*y*]} is less than 0, and the score of the substring pair {*S_db_*[*i*, *i*+*z*], *S_q_*[*j*, *j*+*z*]} exceeds the threshold *T_seed_*, then GHOSTX finds another pair {*S_db_*[*i*+*x*, *i*+*z*], *S_q_*[*j*+*x*, *j*+*z*]} whose score exceeds *T_seed_*. Therefore, GHOSTX examines only those pairs with scores greater than 0. The number of search candidate substrings drastically decreases as they become longer. For example, if GHOSTX checks {A, R} and uses the BLOSUM62 score matrix, the *score* of this pair is −1. Therefore, GHOSTX does not extend the substrings in this pair. Consequently, GHOSTX can find long seeds quickly using these pruning methods. In addition, GHOSTX uses a depth-first search for the implementation of this algorithm to save memory. With a breadth-first search, the depth of the recursion in a seed search is proportional to the exponential of *length_max_*, and thus it is difficult to check all pairs of substrings. However, the depth of recursion in *SeedSearchCore* is *O*(*length_max_*Σ^2^) based on a depth-first search. Therefore, using this depth first search strategy can save memory.

Even when using a binary search, this seed search approach was originally a bottleneck in GHOSTX. To accelerate the process GHOSTX searches parts of seeds using an auxiliary data structure. GHOSTX stores the search results for all substrings whose length is less than 6 on a table before the database search. This process is performed only once, similar to the construction of the database index. In the seed search, GHOSTX can find the search result for a substring without performing a binary search on the suffix array of a database, if the length of the substring is shorter than 6. If we store the search results for longer substrings, we can make the process more efficient. However, the table requires more memory depending on the length of the substring. If the length *length_substring_* of a substring is extended by 1, the size of table increases by O(Σ*^length^_substring_*). Thus, GHOSTX only stores the search results for the substring whose length is less than 6.

### Ungapped Extension and Chain Filtering

Decreasing the number of seeds is critical for the acceleration of a search. However, higher *T_seed_* values cause an increase in the number of significant hits missed, so it is difficult to use high *T_seed_* values without sacrificing sensitivity. Therefore, GHOSTX performs an ungapped extension, which extends seeds without any gaps and excludes low-score extended seeds, after the seed search step, as in BLAST. In the ungapped extension step, GHOSTX uses dropoff termination [4].

**Figure 5:**
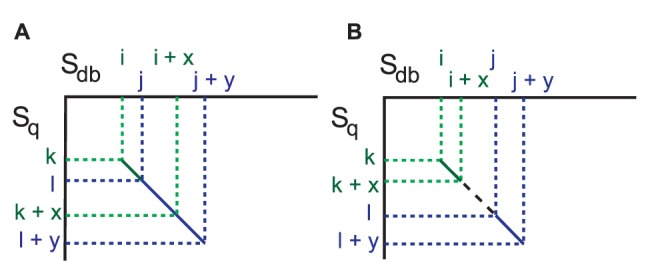
Conditions for reducing seeds in chain filtering.

Some seeds may overlap with others after the seed search and the ungapped extension step. In particular, if there is a sequence highly similar to a query in the database, many seeds that overlap with others are found, and almost identical alignments are often obtained from these overlapped seeds. Thus, it is necessary to merge such overlapped seeds to reduce the number of gapped extensions. Therefore, GHOSTX uses a chain filtering technique. There are two cases in which the seeds are filtered out, as shown in Figure 5. First, if two seeds {*S_db_*[*i*, *i*+*x*], *S_q_*[*k*, *k*+*x*]} and {*S_db_*[*j*, *j*+*y*], *S_q_*[*l*, *l*+*y*]} overlap as shown in Figure 5A, GHOSTX combines these overlapped seeds together into one. Second, if two seeds {*S_db_*[*i*, *i*+*x*], *S_q_*[*k*, *k*+*x*]} and {*S_db_*[*j*, *j*+*y*], *S_q_*[*l*, *l*+*y*]} do not overlap but the score exceeds the dropoff parameter used for the ungapped extension step, as shown in Figure 5B, GHOSTX also merges the overlapped seeds.

### Gapped Extension

Those seeds judged as meaningful by the chain filter are extended with gaps. In the gapped extension, GHOSTX employs dynamic programming and the same heuristics as BLAST. In BLAST gapped extension, the process stops if the score is much lower than the best score, which saves computation time. GHOSTX also employs this technique and uses the same cutoff parameter.

### Database Division

GHOSTX requires a large amount of memory in its homology search. Memory size depends on database size. However, computing systems generally have relatively small memory sizes compared with current database sizes. Therefore, GHOSTX divides a database into several chunks, each of whose size is *l_db_*, before it constructs its indexes. GHOSTX sequentially searches each database chunk, and merges its results with the results of previous chunk searches, when this chunk division is performed before the construction of its database indexes. GHOSTX dramatically reduces working memory requirements using this approach.

### Multithreading Implementation

GHOSTX can be run in multithreading mode. Each query is searched independently, and GHOSTX divides query sequences into several parts. Therefore, each thread independently searches different parts of the query sequences. GHOSTX uses OpenMP for running in multithread mode.

## Results and Discussion

### Datasets and Conditions

To evaluate the performance of our tool, we compared its search sensitivity and computation time to National Center for Biotechnology Information (NCBI) BLASTX (version 2.2.28+), BLAT (version 34 standalone) and RAPSearch (version 2.12). We used the binaries of BLASTX and BLAT downloaded from Web sites. We used RAPSearch compiled with GCC (version 4.3.4) and the –O3 optimizing option. We also compiled GHOSTX using GCC with the –O3 optimizing option and –fopenmp, because GHOSTX can use OpenMP for multithreading. We used a database obtained from KEGG GENES [11], [12] protein sequences as of May 2013. This database contained approximately 10 million protein sequences, with a total size of approximately 3.6 billion residues (3.9 GB). We also used another database obtained from NCBI non-redundant protein sequences (nr) that contained 25 million sequences, approximately 8.6 billion residues (14.8 GB), to check our algorithm’s dependency on database size. For the query sequences, we used 2 query sets: one from human microbiome metagenomic sequences (SRS011098), and the other of soil microbiome metagenomic sequences (SRR444039). SRS011098 was obtained from the Data Analysis and Coordination Center for Human Microbiome Project [13] Web site (http://www.hmpdacc.org/). We used the whole metagenomic shotgun sequencing data from SRS011098. SRR444039 was obtained from the Sequence Read Archive. 10 thousand randomly selected DNA short reads were used from both sets, SRS011098 and SRR444039. We also used 100 thousand randomly selected high quality DNA short reads from SRS011098 to measure multithreading computation time. We performed the analyses on a workstation with two 2.93 GHz Intel Xeon 5670 processors for a total of 12 CPU cores and 54 GB of memory.

### Relationship between GHOSTX Parameters and Sensitivity and Computation Time

GHOSTX has two parameters for its seed search, threshold of the seed search *T_seed_*, and an upper mismatch score *D*. These parameters affect the performance of GHOSTX. Therefore, we first searched for optimal parameters. To determine the best parameters, we used *T_seed_* = 22, 24, 26, 28, 30, 32 and *D* = 1, 4, 7. To evaluate search sensitivity, we used the search results obtained using Smith-Waterman local alignment by SSEARCH [14] as the correct answer. Because the Smith-Waterman algorithm is based on the dynamic programming algorithm and does not use any heuristics, it returns an optimal local alignment. We analyzed the performance of the particular parameter in terms of the fraction of its results that corresponded to the correct answers. When the subject sequences that had the highest score by SSEARCH and each particular method corresponded on each query, the query was deemed correct. Table S1 shows the sensitivity and computation time of each different parameter. As shown in the table, when *T_seed_* is large or *D* is small, the sensitivity of GHOSTX is low and its computing speed is fast. This is because the search space in the seed search is small and the number of seeds is small. However, when *T_seed_* is small or *D* is large, the sensitivity of GHOSTX is high and its computing speed is slow. This is because the search space in the seed search is large and the number of seeds is large. We selected *T_seed_* = 30 and *D* = 4 as default parameters that have a good balance between sensitivity and computation time. We used those parameters in the following evaluations.

### Evaluation of Search Sensitivity

**Figure 6:**
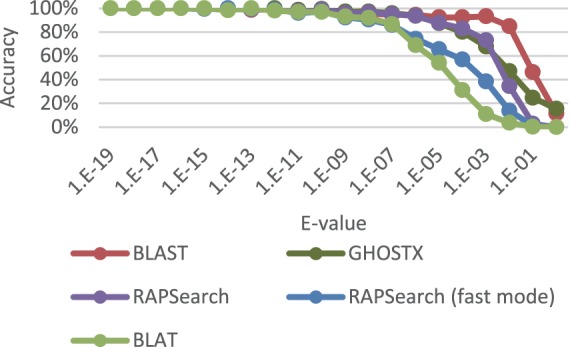
Search sensitivity of each tool with KEGG GENES. The vertical axis shows the percentage of correct answers that correspond to the correct answers for each method. The horizontal axis shows the E-value of the alignments.

To evaluate search sensitivity, we evaluated sensitivity the same way as we evaluated the relationship between GHOSTX seed search parameters and their sensitivity and computation time. To evaluate the software, we executed the BLASTX program with the command line options “-outfmt 6 -comp_based_stats 0”, which instructed the program to output in tabular format, without using composition-based statistics [15], because composition-based statistics are not available in SSEARCH. We used default parameters for the other options. The BLAT program does not include a function to translate DNA reads to protein sequences. Therefore, we translated the DNA reads into protein sequences based on all six potential frames using a standard codon table before executing BLAT. We executed the BLAT program with the command line option “–q = prot –t = prot –out = blast8”, which instructed the program to run the queries and database as protein sequences, and to output data in the BLAST tabular format. We could not execute BLAT when we used nr as a database because our machine has insufficient memory for the execution. Therefore, we only executed BLAT with KEGG GENES. We executed the RAPSearch program with 2 cases. One case used the default options and the other used the command line option “–a T”, which instructed the program to perform a fast mode search. For GHOSTX, we used the following parameters: threshold of the seed search *T_seed_* = 30, upper mismatch score *D* = 4, and size of the database chunk *l_db_* = 2 GB. The other parameters used are the same as BLAST defaults. In Figure 6, GHOSTX shows lower sensitivity than BLASTX, especially for those hits with E-values above 10^−3^. However, alignments with such high E-values are not normally used in most practical analyses anyway, because it is difficult to judge whether the results are merely because of chance. In fact, most research have ignored those hits with such high E-values [1], [2]. Therefore, we think GHOSTX has sufficient search sensitivity for most practical analyses. The sensitivity of GHOSTX is clearly better than that of BLAT and RAPSearch in fast mode, and almost equal to, or better than that of RAPSearch.

### Evaluation of Computation Time

We ran each method with the same commands as for the evaluation of search sensitivity to measure computation time. We used 2 query sets, 10 thousand randomly selected DNA short reads from SRS011098 and from SRR444039, and we used KEGG GENES as our database. Table 1 and Table 2 show the computation time for each program. As shown with each query set, GHOSTX showed accelerations of approximately 153 and 152 times with respect to BLASTX, and approximately 3.5 and 3.5 times with respect to BLAT. Additionally, GHOSTX was approximately 1.6 and 1.5 times faster than RAPSearch, even though GHOSTX showed better search sensitivity than RAPSearch at E-values above 10^−3^. GHOSTX outperforms BLASTX in reducing computation time. The processing time acceleration is caused by the use of a suffix array for its seed search and ungapped extension steps. GHOSTX was slower than RAPSearch in fast mode. However, the sensitivity of RAPSearch in fast mode is clearly lower than GHOSTX.

**Table 1:** Computation time with SRS011098 and KEGG GENES (3.9 GB).

	Computation time (sec.)	Acceleration ratio
GHOSTX	401.9	152.6
RAPSearch	649.5	94.4
RAPSearch in fast mode	91.2	672.2
BLAT	1409.7	43.5
BLAST	61314.1	1.0

The first, second, and third columns show the name of each program, the computation time, and the acceleration in processing speed relative to BLASTX using 1 thread, respectively.

**Table 2:** Computation time with SRR444039 and KEGG GENES (3.9 GB).

	Computation time (sec.)	Acceleration ratio
GHOSTX	362.7	151.8
RAPSearch	553.2	99.5
RAPSearch in fast mode	64.8	849.6
BLAT	1265.3	43.5
BLAST	55045.0	1.0

The first, second, and third columns show the name of each program, the computation time, and the acceleration in processing speed relative to BLASTX using 1 thread, respectively.

We also checked the dependency on the database size for each program by using a larger database. Table 3 and Table 4 show the computation times and accelerations for NCBI nr. GHOSTX showed a better acceleration ratio against BLASTX, as compared with the KEGG GENES database (approximately 165 times and 131 times, respectively). This indicates that these programs can efficiently handle an increase in database size in the future. In contrast to GHOSTX’s acceleration as compared with BLASTX, GHOSTX’s acceleration ratio was 1.5 and 1.4 times as fast as RAPSearch with the larger database, and almost the same when using the smaller KEGG GENES database. Thus, the acceleration ratio of GHOSTX to RAPSearch would not significantly change regardless of the size of a database.

**Table 3:** Computation time with SRS011098 and NCBI nr (14.8 GB).

	Computation time (sec.)	Acceleration ratio
GHOSTX	1020.1	165.2
RAPSearch	1564.4	107.7
RAPSearch in fast mode	223.8	752.8
BLAT	N/A	N/A
BLAST	168488.0	1.0

The first, second, and third columns show the name of each program, the computation time, and the acceleration in processing speed relative to BLASTX using 1 thread, respectively.

**Table 4:** Computation time with SRR444039 and NCBI nr (14.8 GB).

	Computation time (sec.)	Acceleration ratio
GHOSTX	1003.5	130.8
RAPSearch	1404.1	93.4
RAPSearch in fast mode	223.8	586.2
BLAT	N/A	N/A
BLAST	131213.3	1.0

The first, second, and third columns show the name of each program, the computation time, and the acceleration in processing speed relative to BLASTX using 1 thread, respectively.

We measured the computation time of preprocessing, including database indexing, for GHOSTX, BLAST and RAPSearch. Table 5 shows the computation time for preprocessing. Preprocessing in GHOSTX requires computation time almost equal to RAPSearch. However, homology search computation time is generally much larger than that required for the database construction phase when a huge amount of DNA reads obtained from next-generation sequencers are processed. Moreover, preprocessing is only performed when a database is updated. Therefore, we think preprocessing is not a problem in practice.

**Table 5:** Computation time of the preprocessing including indexing with KEGG GENES (3.9 GB) and NCBI nr (14.8 GB).

	Computation time with KEGG GENES (sec.)	Computation time with NCBI nr (sec.)
GHOSTX	1589.2	4415.2
RAPSearch	1914.2	4210.5
BLAST	637.6	1678.9

The first, second, and third columns show the name of each program, the computation time with KEGG GENES, and the computation time with NCBI nr.

### Evaluation of Multithreading Computation Time

**Figure 7:**
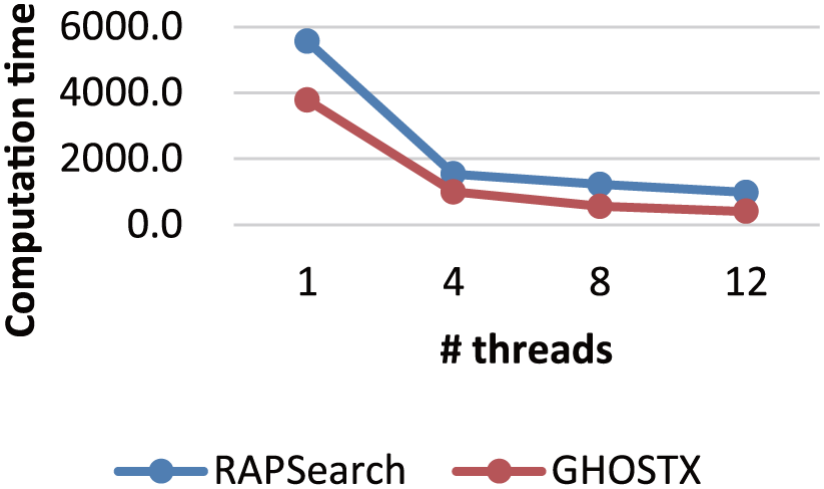
Computation times with multithreading.

To evaluate multithreading computation time, we ran GHOSTX with *T_seed_* = 30 and *D* = 4, and RAPSearch with its default options except for the multithreading option. We used 100 thousand randomly selected DNA short reads from SRS011098 as queries and KEGG GENES as the database, because 10 thousand randomly selected DNA short reads were too small of a sample to measure correct computation time. Figure 7 shows the computation time for each program with 1, 4, 8, and 12 threads. As shown, GHOSTX sufficiently scales with multithreading. GHOSTX shows an acceleration of approximately 9.4 times with 12 threads as opposed to GHOSTX with 1 thread.

### Evaluation of Memory Size

While GHOSTX can search for homologues more efficiently than BLAST, GHOSTX requires more memory. GHOSTX uses approximately 18 GB of memory for constructing the indexes of a typical database, and approximately 13 GB for the homology search itself, when a 2 GB database chunk is used. However, using a smaller database chunk size can decrease the amount of memory required. Table 6 shows the relationships between the amount of memory required to construct the indexes and homology search versus the size of a database chunk. The required memory size of GHOSTX is almost linearly increased in proportion to the size of a database chunk. If a database is divided into more chunks, the required memory size becomes smaller accordingly. Therefore, with smaller database chunk sizes, GHOSTX can be executable even on a general PC. Of course, there is a trade-off between database chunk size and search speed. Homology search computation times increase as the size of a database chunk becomes smaller. This is so because the same suffix array search has to be performed for each respective chunk, and the number of suffix array searches increases as a result. However, the situation is not dire; as shown in Table 7, the search speed of GHOSTX with 512 MB chunks is approximately 20% slower than that with 2 GB chunks. The maximum size of a database chunk is 2 GB in GHOSTX, because the maximum size of a 32 bit integer is 2 GB.

**Table 6:** Comparison with memory size for KEGG GENES (3.9 GB) of each size of the database chunks.

Chunk size	Memory size for constructing index (GB)	Memory size for homology search (GB)
512 MB	4.6	4.2
1 GB	9.2	7.2
2 GB	18.2	13.3

The first, second, and third columns show the size of the database chunk, the used memory size for constructing index (GB), and the used memory size for homology search (GB).

**Table 7:** Comparison with Computation time for KEGG GENES (3.9 GB) of each size of the database chunks.

Chunk size	Computation time (sec.)	Acceleration ratio
512 MB	526.9	0.8
1 GB	452.7	0.9
2 GB	401.9	1.0

The first, second, and third columns show the size of the database chunk, the computation time, and the acceleration in processing speed relative to GHOSTX with 2 GB database chunks, respectively.

## Conclusions

We have developed an efficient algorithm for performing sequence homology searches, and have implemented it as GHOSTX. GHOSTX has sufficient search sensitivity for practical analyses. It uses an extremely efficient seed search algorithm, employing database and query suffix arrays, to achieve a well over 100 times faster sequence homology search than BLASTX. GHOSTX is also almost 1.4–1.6 times faster than RAPSearch, which is one of the fastest homology search tools available, even though GHOSTX is slightly more accurate. Currently, sequencing technology continues to improve, increasingly producing larger and larger quantities of data. This explosion of sequence data makes computational analysis with contemporary tools more difficult. We offer this tool as a potential solution to the problem.

## Supporting Information

Table S1 **Relationship between GHOSTX parameters and sensitivity and computation time.** The first, second, and third columns show the parameter, the sensitivity, and the computation time. The sensitivity is calculated as the ratio of correctly searched queries whose E-values<10^−3^.
(DOC)
