# Supplementary material for: GHOSTX: An Improved Sequence Homology Search Algorithm Using a Query Suffix Array and a Database Suffix Array
Source: PLoS One. 2014 Aug 6;9(8):e103833. doi: 10.1371/journal.pone.0103833 (PMC4123905; doi:10.1371/journal.pone.0103833)
Supplement: Table S1 — Relationship between GHOSTX parameters and sensitivity and computation time. The first, second, and third columns show the parameter, the sensitivity, and the computation time. The sensitivity is calculated as the ratio of correctly searched queries whose E-values<10−3. (DOC) [file pone.0103833.s001.doc]

**Table S1. Relationship between GHOSTX parameters and sensitivity and computation time.** The first, second, and third columns show the parameter, the sensitivity, and the computation time. The sensitivity is calculated as the ratio of correctly searched queries whose E-values < 10^-3^.

| GHOSTX parameters | Sensitivity | Computation time (sec.) |
| --- | --- | --- |
| *T_seed_* = 22*, D* = 1 | 0.960 | 615.0 |
| *T_seed_* = 24*, D* = 1 | 0.957 | 474.4 |
| *T_seed_* = 26*, D* = 1 | 0.952 | 316.1 |
| *T_seed_* = 28*, D* = 1 | 0.940 | 199.4 |
| *T_seed_* = 30*, D* = 1 | 0.925 | 151.2 |
| *T_seed_* = 32*, D* = 1 | 0.913 | 119.5 |
| *T_seed_* = 22*, D* = 4 | 0.977 | 2099.0 |
| *T_seed_* = 24*, D* = 4 | 0.975 | 1284.4 |
| *T_seed_* = 26*, D* = 4 | 0.972 | 779.9 |
| *T_seed_* = 28*, D* = 4 | 0.966 | 518.4 |
| *T_seed_* = 30*, D* = 4 | 0.959 | 401.9 |
| *T_seed_* = 32*, D* = 4 | 0.949 | 344.1 |
| *T_seed_* = 22*, D* = 7 | 0.980 | 7066.6 |
| *T_seed_* = 24*, D* = 7 | 0.980 | 4312.9 |
| *T_seed_* = 26*, D* = 7 | 0.979 | 3035.2 |
| *T_seed_* = 28*, D* = 7 | 0.978 | 2472.6 |
| *T_seed_* = 30*, D* = 7 | 0.973 | 2060.8 |
| *T_seed_* = 32*, D* = 7 | 0.968 | 2101.9 |
